# Neuropsychological Outcomes in Individuals With Type 1 and Type 2 Diabetes

**DOI:** 10.3389/fendo.2022.834978

**Published:** 2022-03-04

**Authors:** Nathaniel M. Putnam, Evan L. Reynolds, Mousumi Banerjee, Kara Mizokami-Stout, Dana Albright, Joyce Lee, Rodica Pop-Busui, Eva L. Feldman, Brian C. Callaghan

**Affiliations:** ^1^ Department of Biostatistics, University of Michigan, Ann Arbor, MI, United States; ^2^ Department of Neurology, University of Michigan, Ann Arbor, MI, United States; ^3^ Department of Internal Medicine, Division of Metabolism, Endocrinology, and Diabetes, University of Michigan, Ann Arbor, MI, United States; ^4^ Department of Pediatrics, Division of Pediatric Psychology, University of Michigan, Ann Arbor, MI, United States; ^5^ Department of Pediatrics, Division of Pediatric Endocrinology, University of Michigan, Ann Arbor, MI, United States

**Keywords:** diabetes mellitus, regress analysis, big data and analytics, mental health, diabetes - quality of life

## Abstract

**Objective:**

To determine the prevalence of neuropsychological outcomes in individuals with type 1 diabetes compared to individuals with type 2 diabetes or without diabetes, and to evaluate the association of diabetes status and microvascular/macrovascular complications with neuropsychological outcomes.

**Patients and Methods:**

We used a nationally representative healthcare claims database of privately insured individuals (1/1/2001-12/31/2018) to identify individuals with type 1 diabetes. Propensity score matching was used as a quasi-randomization technique to match type 1 diabetes individuals to type 2 diabetes individuals and controls. Diabetes status, microvascular/macrovascular complications (retinopathy, neuropathy, nephropathy, stroke, myocardial infarction, peripheral vascular disease, amputations), and neuropsychological outcomes (mental health, cognitive, chronic pain, addiction, sleep disorders) were defined using ICD-9/10 codes. Logistic regression determined associations between diabetes status, microvascular/macrovascular complications, and neuropsychological outcomes.

**Results:**

We identified 184,765 type 1 diabetes individuals matched to 524,602 type 2 diabetes individuals and 522,768 controls. With the exception of cognitive disorders, type 2 diabetes individuals had the highest prevalence of neuropsychological outcomes, followed by type 1 diabetes, and controls. After adjusting for the presence of microvascular/macrovascular complications, type 1 diabetes was not significantly associated with a higher risk of neuropsychological outcomes; however, type 2 diabetes remained associated with mental health, cognitive, and sleep disorders. The presence of microvascular/macrovascular complications was independently associated with each neuropsychological outcome regardless of diabetes status.

**Conclusion:**

Microvascular/macrovascular complications are associated with a high risk of neuropsychological outcomes regardless of diabetes status. Therefore, preventing microvascular and macrovascular complications will likely help reduce the likelihood of neuropsychological outcomes either as the result of similar pathophysiologic processes or by preventing the direct and indirect consequences of these complications. For individuals with type 2 diabetes, risk factors beyond complications (such as obesity) likely contribute to neuropsychological outcomes.

## Introduction

Individuals with type 1 diabetes are at an increased risk for a number of microvascular and macrovascular complications, including retinopathy, neuropathy, nephropathy, stroke, myocardial infarction, peripheral vascular disease, and amputations (1). These complications result in substantial mortality, morbidity, and reduced quality of life (2). In contrast to these microvascular and macrovascular complications of diabetes, much less is known about the neuropsychological outcomes of type 1 diabetes including mental health, cognitive, chronic pain, addiction, and sleep disorders.

The current literature supports a higher burden of most neuropsychological outcomes in individuals with type 1 diabetes relative to the general population. The most well studied neuropsychological outcomes of type 1 diabetes are mental health disorders, specifically depression and anxiety. A systematic review found that the prevalence of depression and anxiety for individuals with type 1 diabetes is nearly three times that of the general population (3). Similarly, a systematic review found that individuals with type 1 diabetes had impaired cognitive function across broad categories including visual-spatial ability and memory (4). Multiple studies have also documented elevated rates of pain in adults and adolescents with type 1 diabetes (5, 6). In contrast to other neuropsychological outcomes, a systematic review found that there were similar rates of substance use between young adults with and without type 1 diabetes (7). Lastly, a meta-analysis found that children with type 1 diabetes get less sleep, adults with type 1 diabetes have lower quality sleep, and that type 1 diabetes is associated with higher rates of obstructive sleep apnea compared to the general population (8). However, these studies have four key limitations. Namely, their sample sizes were relatively small, they rarely investigated the role of microvascular and macrovascular complications, they were often focused on young individuals, and/or they lacked a control group (3–8). Furthermore, no previous study comprehensively evaluated the full spectrum of neuropsychological outcomes and few compared these complications for individuals with type 1 *vs*. type 2 diabetes. Our study fills these gaps in the literature using a large, nationally representative sample of privately insured individuals in the US.

Our objective was to describe and compare the prevalence of neuropsychological outcomes for individuals with type 1 diabetes, type 2 diabetes, and individuals without diabetes and to explore the independent effects of diabetes status and microvascular/macrovascular complications on these neuropsychological outcomes.

## Methods

### Population

We utilized the de-identified Optum Analytics database, which consists of detailed medical and pharmaceutical claims on tens of millions of insured individuals from 2001-2018. As the largest claims data repository in the United States, the demographic makeup of the Optum Analytics database closely matches those of the privately insured population. Using a validated ICD-9/ICD-10 code definition (9), we identified individuals with type 1 diabetes (250.x1, 250.x3, E10.xx) and type 2 diabetes (250.x0, 250.x2, E11.xx). For individuals with both type 1 and type 2 diabetes diagnosis codes, greater than 50% of one type of code determined individual diabetes type (9); this definition has sensitivity of 63% and positive predictive value of 94% for identifying individuals with type 1 diabetes, and a sensitivity of 100% and positive predictive value of 90% for identifying individuals with type 2 diabetes. The population was restricted to the first enrollment period for individuals with complete demographic and socioeconomic information.

### Neuropsychological Outcomes

Neuropsychological outcomes were defined by aggregating across diagnoses specific to mental health, cognitive, chronic pain, addiction, and sleep disorders. These conditions were defined using ICD-9/ICD-10 codes (**Supplemental Table S1**) from the period of follow-up after diabetes diagnosis, or an analogous portion of follow-up in individuals without diabetes. Specifically, mental health disorders were determined as whether individuals had a diagnosis of anxiety, Attention Deficit Hyperactivity Disorder (ADHD), adjustment disorder, eating disorder, depression, Post-Traumatic Stress Disorder (PTSD), or other behavioral and emotional disorders (10–12). Cognitive disorders were determined as whether individuals had a diagnosis of dementia, mild cognitive impairment, Alzheimer’s, or vascular dementia (13–15). Addiction disorders were determined as whether individuals had a diagnosis indicating dependence on alcohol, opioids, cocaine, sedatives, hallucinogens, nicotine, inhalant, other stimulants, and, other psychoactive and non-psychoactive chemicals (16). Chronic pain disorders were determined as whether individuals had a diagnosis of chronic pain based on a previously validated definition that included postherpetic neuralgia, trigeminal neuralgia, HIV-associated pain, stroke-associated pain, chronic pain syndrome, lumbar radiculopathy, complex regional pain syndrome, spinal cord injury, surgically-induced pain, phantom limb, cervical radiculopathy, multiple sclerosis-associated pain, fibromyalgia, osteoarthritis, low back pain, migraine, rheumatoid arthritis, ankylosing spondylitis, psoriatic arthropathy, cancer pain, irritable bowel syndrome, painful bladder syndrome, and interstitial cystitis (17). In addition to the above conditions, chronic headache, chronic fatigue syndrome, temporomandibular joint disorder, and chronic pelvic pain (endometriosis or vulvodynia) were included as chronic pain conditions. Sleep disorders were determined as whether individuals had a diagnosis of insomnia, hypersomnia, sleep apnea, circadian rhythm disorders, or other sleep disorders (18).

### Microvascular and Macrovascular Complications of Diabetes

We used ICD-9/ICD-10 codes to determine if individuals had microvascular or macrovascular complications of diabetes during the period of follow-up after diabetes diagnosis, or an analogous portion of total follow-up in individuals without diabetes (**Supplemental Table S1**). Microvascular complications included retinopathy, neuropathy and nephropathy (19, 20). Macrovascular complications included stroke, myocardial infarction, and peripheral vascular disease (14, 20, 21). Amputation was also included as a complication, but was not included as a microvascular or macrovascular complication since it results from both mechanisms.

### Matching

Individuals with type 1 diabetes were matched to individuals with type 2 diabetes and non-diabetic controls stratified by age (0-20, 20-40, 40-60, 60+) using propensity scores within a caliper of 0.10 for individuals age 0-20 and within 0.01 for individuals age 20+ (22). Propensity scores were calculated based on individual age at study entry, sex, race/ethnicity, geographic region, education level, net worth, insurance plan type, high deductible health plan status, modified Charlson Comorbidity Index, starting year of enrollment, and length of follow-up. The modified version of the Charlson Comorbidity Index consisted of conditions that did not overlap with study outcomes. Specifically, the modified Charlson Comorbidity Index included congestive heart failure, chronic pulmonary disease, connective tissue disease, peptic ulcer disease, mild liver disease, paraplegia and hemiplegia, renal disease excluding diabetic nephropathy, cancer, liver disease, metastatic carcinoma, and HIV (23). Individuals with diabetes were also matched based on length of pre-diagnosis and post-diagnosis enrollment.

Based on the availability of well-matched controls without diabetes, each individual age 0-40 with type 1 diabetes was matched to 1 individual with type 2 diabetes and then independently matched with 1 non-diabetic control. For those age>40, each individual with type 1 diabetes was matched to 4 individuals with type 2 diabetes and then independently matched with 4 non-diabetic controls.

### Statistical Analysis

Descriptive statistics were used to characterize the matched individuals, stratified by age. We used a Cochrane-Mantel-Haenszel test to compare the prevalence of each neuropsychological outcome and each microvascular/macrovascular complication stratified by diabetes type and age. Multivariable logistic regression was used to assess the association between diabetes status, microvascular and macrovascular complications, and each neuropsychological outcome (mental health, cognitive, chronic pain, addiction, and sleep disorders). Specifically, for each of the 5 outcomes, we fit a model as a function of diabetes status (type 1 diabetes *vs*. type 2 diabetes *vs*. non-diabetic controls) and presence of any microvascular or macrovascular complications, independent of diabetes status, stratified by age. Wald Tests were used to determine statistical significance of the effects of diabetes status and presence of microvascular or macrovascular complications on neuropsychological outcomes. Since very few individuals between the ages of 0-40 had cognitive disorders, we did not fit logistic regression models for those age strata.

To investigate the effects of distinct complications on each neuropsychological outcome, we fit additional models, first separating complications into microvascular, macrovascular, and amputations, and then another model including each specific complication as an individual covariate (retinopathy, neuropathy, nephropathy, stroke, myocardial infarction, peripheral vascular disease, and amputations).

For all hypothesis testing, statistical significance was determined using a P-value threshold of 0.05.

All analyses were performed using SAS version 9.4 (Cary, NC, USA).

This study was considered exempt by the Institutional Review Board of the University of Michigan.

## Results

### Demographic and Socioeconomic Information of Matched Individuals

We identified 16,179 individuals aged 0-20 and 55,293 individuals aged 20-40 with type 1 diabetes that were each matched to 1 individual with type 2 diabetes and 1 individual without diabetes. Similarly, we identified 63,777 individuals aged 40-60 and 49,516 individuals age 60+ with type 1 diabetes that were each matched to 4 individuals with type 2 diabetes and 4 individuals without diabetes.

Descriptive statistics of the matched individuals’ demographic, socioeconomic, and insurance plan information are presented in **Table 1**. Within age strata, individuals were closely matched in all characteristics. In individuals with type 1 diabetes, the mean follow-up length after diabetes diagnosis was 2.41 years (SD 2.94) for individuals ages 0-20, 1.56 years (SD 2.13) for individuals ages 20-40, 2.07 years (SD 2.67) for individuals ages 40-60, and 2.48 years (SD 3.04) for individuals ages 60+. In individuals with type 2 diabetes, the mean follow-up length after diabetes diagnosis was 2.42 years (SD 2.60) for individuals ages 0-20, 1.59 years (SD 1.93) for individuals ages 20-40, 2.07 years (SD 2.67) for individuals ages 40-60, and 2.47 years (SD 2.91) for individuals ages 60+. Matched individuals were roughly 50% female (within 3% in each group). Approximately 10% of matched individuals were black, except in the 60+ age strata, where approximately 15% of individuals were black. Individuals aged 0-40 were 10-12% Hispanic, while individuals age 40+ were 8-9% Hispanic.

**Table 1:** Demographics of matched cohort, stratified by age and diabetes type.

			age 0-20 (N=48,537)				age 20-40 (N=165,877)				
Variable			Type 1 Diabetes (n=16,179)	Type 2 Diabetes (n=16,179)		No Diabetes (n=16,179)	Type 1 Diabetes (n=55,293)		Type 2 Diabetes (n=55,293)		No Diabetes (n=55,291)
Age		Mean years (SD)	14.1 (4.52)	14. (5.45)		14.1 (4.95)	31.0 (5.60)		30. (5.63)		30.9 (5.61)
Gender (%)		Female	52.0%	52.2%		51.9%	49.4%		49.8%		49.0%
		Male	48.0%	47.8%		48.1%	50.6%		50.2%		51.0%
Race (%)		Asian	3.2%	3.0%		3.4%	2.8%		3.0%		2.9%
		Black	10.7%	10.3%		10.7%	10.1%		10.0%		10.2%
		Hispanic	12.6%	12.6%		12.4%	10.2%		10.5%		10.8%
		White	73.5%	74.1%		73.5%	76.9%		76.5%		76.1%
State (%)		IL, IN, MI, OH, WI	16.2%	16.6%		16.2%	16.1%		15.9%		15.3%
		AL, KY, MS, TN	4.8%	4.7%		5.0%	5.6%		5.6%		5.6%
		NJ, NY, PA	9.4%	9.4%		9.4%	8.7%		9.2%		9.1%
		AZ, CO, ID, MT, NV, NM, UT, WY	7.2%	7.2%		7.2%	8.7%		8.6%		9.0%
		CT, ME, MA, NH, RI, VT	3.3%	3.4%		3.2%	2.8%		2.9%		3.1%
		AK, CA, HI, OR, WA	8.8%	8.6%		8.6%	8.2%		8.2%		8.6%
		DE, DC, FL, GA, MD, NC, SC, VA, WV	26.8%	26.7%		27.5%	25.2%		25.1%		25.0%
		IA, KS, MN, MO, NE, ND, SD	7.9%	7.8%		7.4%	10.5%		10.7%		10.3%
		AR, LA, OK, TX	15.5%	15.4%		15.3%	14.1%		13.8%		13.9%
Education Level (%)		Less than 12th Grade	0.6%	0.6%		0.7%	0.7%		0.8%		0.7%
		High School Diploma	29.9%	30.1%		30.2%	27.8%		27.6%		27.4%
		Less than bachelor’s degree	52.5%	52.3%		51.9%	53.7%		53.2%		53.2%
		Bachelor’s degree Plus	17.0%	17.0%		17.2%	17.8%		18.4%		18.7%
Net Worth (%)		<$25K	27.4%	27.2%		26.6%	33.2%		32.6%		32.8%
		$25K-$149K	24.1%	24.3%		24.1%	27.2%		27.0%		26.7%
		$150K-$249K	12.0%	11.8%		12.2%	11.7%		11.7%		11.8%
		$250K-$499K	16.9%	17.1%		17.0%	14.5%		14.7%		15.1%
		$500K+	19.5%	19.6%		20.1%	13.5%		14.1%		13.6%
Insurance Provider (%)		Exclusive Provider Organization	13.6%	13.7%		13.6%	12.2%		12.1%		12.6%
		Health Maintenance Organization	18.5%	18.1%		18.2%	20.3%		20.3%		19.1%
		Indemnity	0.0%	0.0%		0.0%	0.1%		0.1%		0.1%
		Other	0.1%	0.1%		0.1%	0.8%		0.9%		0.8%
		Point of Service	60.8%	61.1%		61.2%	58.1%		58.0%		58.8%
		Preferred Provider Organization	6.9%	7.0%		6.8%	8.5%		8.7%		8.6%
Customer Driven Health Plan Type (%)		Health Reimbursement Arrangement	6.0%	5.9%		6.1%	4.8%		4.9%		5.0%
		Health Savings Account	9.3%	9.4%		9.2%	7.5%		7.4%		8.0%
Micro/Macrovascular Complication		Any Complication (%)	7.8%	4.4%		0.6%	22.2%		8.9%		1.6%
		Retinopathy (%)	3.3%	0.5%		0.0%	12.9%		1.8%		0.0%
		Neuropathy (%)	1.4%	1.1%		0.2%	5.3%		2.7%		0.6%
		Nephropathy (%)	2.9%	1.6%		0.2%	7.8%		3.2%		0.4%
		Myocardial Infarction (%)	0.1%	0.1%		0.0%	0.4%		0.3%		0.1%
		Stroke (%)	0.1%	0.2%		0.1%	0.4%		0.4%		0.1%
		Peripheral Vascular Disease (%)	0.8%	0.8%		0.1%	2.5%		1.6%		0.4%
		Amputation (%)	0.4%	0.5%		0.1%	1.3%		0.9%		0.2%
Modified Charlson Comorbidity Score		Mean score (SD)	0.1 (0.34)	0.1 (0.35)		0.11 (0.35)	0.14 (0.42)		0.15 (0.4)		0.14 (0.41)
Years of Follow-up		Mean years (SD)	4.50 (3.77)	4.51 (3.54)		4.52 (3.78)	3.05 (2.93)		3.09 (2.80)		3.06 (3.04)
Years of Follow-up Pre-diabetes diagnosis)		Mean years (SD)	2.08 (2.31)	2.08 (2.20)		N/A	1.48 (1.)		1.50 (1.83)		N/A
Years of Follow-up Post-diabetes diagnosis		Mean years (SD)	2.41 (2.94)	2.42 (2.60)		N/A	1.56 (2.13)		1.59 (1.93)		N/A
			**age 40-60 (N=573,874)**				**age 60+ (N=443,847)**				
**Variable**			**Type 1 Diabetes (n=63,777)**	**Type 2 Diabetes (n=255,066)**	**No Diabetes (n=255,031)**		**Type 1 Diabetes (n=49,516)**	**Type 2 Diabetes (n=198,064)**		**No Diabetes (n=196,267)**	
Age	Mean years (SD)		50. (5.65)	50.4 (5.64)	50.3 (5.64)		71.3 (7.42)	71.3 (7.34)		71.0 (7.15)	
Gender (%)	Female		47.8%	47.7%	47.9%		52.6%	52.6%		52.3%	
	Male		52.2%	52.3%	52.1%		47.4%	47.4%		47.7%	
Race (%)	Asian		2.2%	2.3%	2.3%		2.8%	2.8%		3.0%	
	Black		10.8%	11.0%	11.0%		14.6%	14.5%		14.4%	
	Hispanic		8.2%	8.3%	8.4%		8.8%	8.8%		9.1%	
	White		78.8%	78.5%	78.3%		73.8%	73.9%		73.5%	
State (%)	IL, IN, MI, OH, WI		15.2%	15.2%	15.2%		13.3%	13.2%		13.0%	
	AL, KY, MS, TN		5.9%	5.8%	5.8%		5.6%	5.6%		5.5%	
	NJ, NY, PA		8.4%	8.4%	8.5%		11.6%	11.7%		11.1%	
	AZ, CO, ID, MT, NV, NM, UT, WY		7.4%	7.5%	7.5%		7.2%	7.3%		7.9%	
	CT, ME, MA, NH, RI, VT		3.5%	3.5%	3.6%		5.6%	5.7%		5.4%	
	AK, CA, HI, OR, WA		8.4%	8.3%	8.4%		10.5%	10.5%		10.6%	
	DE, DC, FL, GA, MD, NC, SC, VA, WV		28.2%	28.4%	27.8%		28.6%	28.5%		27.5%	
	IA, KS, MN, MO, NE, ND, SD		10.1%	10.1%	9.9%		7.7%	7.7%		8.5%	
	AR, LA, OK, TX		12.7%	12.9%	13.0%		9.6%	9.4%		10.1%	
Education Level (%)	Less than 12th Grade		0.8%	0.8%	0.8%		1.3%	1.3%		1.3%	
	High School Diploma		30.8%	30.9%	31.0%		36.0%	35.9%		36.0%	
	Less than bachelor’s degree		51.4%	51.0%	51.1%		49.7%	49.9%		49.6%	
	Bachelor’s degree Plus		17.1%	17.3%	17.2%		13.0%	12.9%		13.0%	
Net Worth (%)	<$25K		20.0%	20.1%	20.2%		18.4%	18.2%		18.6%	
	$25K-$149K		22.7%	22.6%	22.7%		21.2%	21.1%		21.4%	
	$150K-$249K		13.5%	13.4%	13.8%		13.7%	13.8%		14.0%	
	$250K-$499K		21.2%	21.1%	20.9%		21.1%	21.2%		21.2%	
	$500K+		22.7%	22.7%	22.4%		25.6%	25.7%		24.9%	
Insurance Provider (%)	Exclusive Provider Organization		10.2%	10.3%	10.5%		2.6%	2.6%		2.7%	
	Health Maintenance Organization		23.4%	23.5%	22.8%		34.4%	34.2%		35.3%	
	Indemnity		0.2%	0.2%	0.2%		3.8%	3.8%		3.9%	
	Other		4.6%	4.6%	4.4%		33.7%	34.0%		32.0%	
	Point of Service		52.4%	52.2%	52.9%		14.1%	14.1%		14.6%	
	Preferred Provider Organization		9.2%	9.2%	9.2%		11.4%	11.4%		11.5%	
Customer Driven Health Plan Type (%)	Health Reimbursement Arrangement		4.4%	4.5%	4.5%		1.2%	1.2%		1.2%	
	Health Savings Account		7.2%	7.2%	7.4%		1.9%	1.8%		2.0%	
Micro/Macrovascular Complication	Any Complication (%)		38.6%	21.9%	6.9%		53.6%	46.6%		25.4%	
	Retinopathy (%)		21.2%	5.0%	0.0%		17.4%	7.8%		0.1%	
	Neuropathy (%)		12.5%	7.9%	2.1%		15.5%	13.6%		4.7%	
	Nephropathy (%)		13.6%	7.3%	1.9%		23.7%	22.7%		11.9%	
	Myocardial Infarction (%)		2.0%	1.8%	0.9%		5.4%	5.2%		3.5%	
	Stroke (%)		1.7%	1.7%	0.9%		6.1%	6.2%		4.6%	
	Peripheral Vascular Disease (%)		9.1%	6.2%	2.1%		22.9%	20.3%		11.1%	
	Amputation (%)		3.9%	2.4%	0.5%		6.2%	4.8%		1.8%	
Modified Charlson Comorbidity Score	Mean score (SD)		0.32 (0.6)	0.3 (0.64)	0.32 (0.6)		0.64 (0.90)	0.65 (0.89)		0.65 (0.89)	
Years of Follow-up	Mean years (SD)		3.8 (3.57)	3.87 (3.35)	3.85 (3.53)		4.21 (3.85)	4.20 (3.57)		4.10 (3.78)	
Years of Follow-up Pre-diabetes diagnosis)	Mean years (SD)		1.79 (2.13)	1.78 (2.07)	N/A		1.7 (2.07)	1.73 (1.94)		N/A	
Years of Follow-up Post-diabetes diagnosis	Mean years (SD)		2.07 (2.67)	2.08 (2.47)	N/A		2.48 (3.04)	2.47 (2.91)		N/A	

N/A, Not Applicable.

### Neuropsychological Outcomes

**Figure 1:**
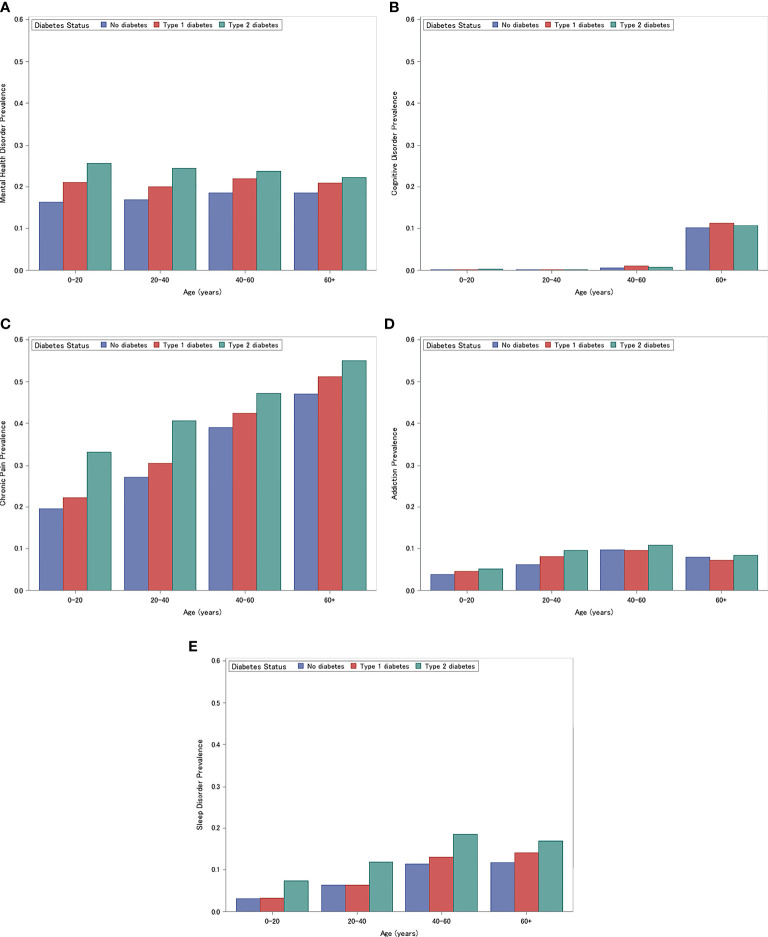
Prevalence of Mental Health Disorders **(A)** Cognitive Disorders **(B)** Chronic Pain **(C)** Addiction **(D)** Sleep **(E)**. **(A)** Prevalence of Mental Health Disorders for individuals with type 1 diabetes, type 2 diabetes, and without diabetes, stratified by age (0-20, 20-40, 40-60, 60+ years). **(B)** Prevalence of Cognitive Disorders for individuals with type 1 diabetes, type 2 diabetes, and without diabetes, stratified by age (0-20, 20-40, 40-60, 60+). **(C)** Prevalence of Chronic Pain for individuals with type 1 diabetes, type 2 diabetes, and without diabetes, stratified by age (0-20, 20-40, 40-60, 60+). **(D)** Prevalence of Addiction for individuals with type 1 diabetes, type 2 diabetes, and without diabetes, stratified by age (0-20, 20-40, 40-60, 60+). **(E)** Prevalence of Sleep Disorders for individuals with type 1 diabetes, type 2 diabetes, and without diabetes, stratified by age (0-20, 20-40, 40-60, 60+).

The unadjusted prevalence of each neuropsychological condition is presented in **Figures 1A–E**. Across all neuropsychological outcomes except cognitive disorders, individuals with type 2 diabetes had the highest prevalence, followed by individuals with type 1 diabetes and then individuals without diabetes (each Cochrane-Mantel-Haenszel Test P<.001). For cognitive disorders, individuals with type 1 diabetes had a higher prevalence than individuals with type 2 diabetes (each Cochrane-Mantel-Haenszel Test P<.001). In each age strata, chronic pain was the most prevalent condition, followed by mental health, sleep, addiction, and cognitive disorders. Cognitive disorders were rare in all age groups except in those greater than 60 years old.

### Microvascular and Macrovascular Complications

**Figure 2:**
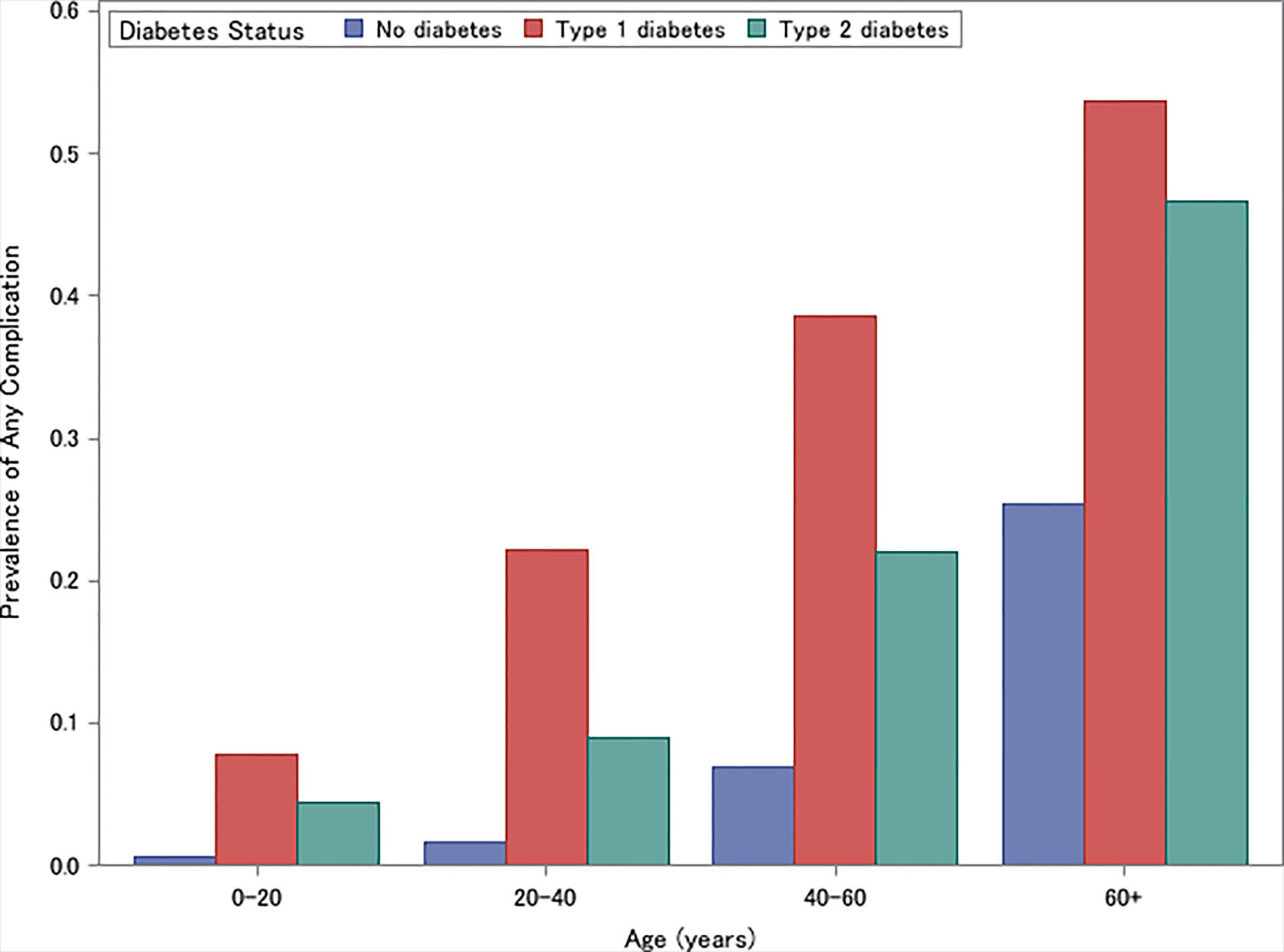
Prevalence of Microvascular or Macrovascular Complications for individuals with type 1 diabetes, type 2 diabetes, and without diabetes, stratified by age (0-20, 20-40, 40-60, 60+).

The unadjusted prevalence of microvascular and macrovascular complications is presented in **Figure 2**. Individuals with type 1 diabetes had the highest prevalence of microvascular and macrovascular complications (ages 0-20: 7.8%, ages 20-40: 22.2%, ages 40-60: 38.6%, ages 60+: 53.6%), followed by individuals with type 2 diabetes (ages 0-20: 4.4%, ages 20-40: 8.9%, ages 40-60: 21.9%, ages 60+: 46.6%) and non-diabetic controls (ages 0-20: 0.6%, ages 20-40: 1.6%, ages 40-60: 6.9%, ages 60+: 25.4%) (Cochrane-Mantel-Haenszel Test: P<.001) (**Figure 2**). This trend was consistent for each individual complication and age strata (all P<.001).

### Mental Health Disorders

The results of the mental health disorder models are presented in **Table 2**. Across age strata and after adjusting for the presence of microvascular/macrovascular complications, individuals with type 2 diabetes (ages 0-20: OR 1.31, 95% CI: 1.28-1.35; ages 20-40: OR 1.24, 95% CI 1.22-1.26; ages 40-60: OR 1.11, 95% CI: 1.10-1.12; ages 60+: OR 1.01, 95% CI: 1.01-1.02) and younger individuals with type 1 diabetes (ages 0-20: OR 1.14, 95% CI: 1.10-1.17; ages 20-40: OR 1.04, 95% CI 1.03-1.06) had significantly higher odds of mental health disorders compared to non-diabetic controls (**Table 2**). In contrast, older individuals with type 1 diabetes had significantly lower odds of mental health disorders compared to non-diabetic controls (ages 40-60: OR 0.98, 95% CI: 0.97, 0.99; ages 60+: OR 0.94, 95% CI: 0.93-0.95). In all age strata, individuals with type 2 diabetes also had significantly higher odds of mental health disorders compared to individuals with type 1 diabetes. After adjusting for diabetes status, the effects of microvascular and macrovascular complications were independently associated with an increased odds of mental health disorders (ages 0-20: OR 1.37, 95% CI: 1.28-1.46; ages 20-40: OR 1.31, 95% CI 1.28-1.34; ages 40-60: OR 1.43, 95% CI: 1.41-1.44; ages 60+: OR 1.61, 95% CI: 1.60-1.63).

**Table 2:** The association between diabetes status and microvascular/macrovascular complications and neuropsychological outcomes stratified by age.

Model Outcome	Covariate	Ages 0-20 OR (95% CI)	Ages 20-40 OR (95% CI)	Ages 40-60 OR (95% CI)	Ages 60 + OR (95% CI)
Mental Health Disorder	Type 1 Diabetes (reference: no diabetes)	1.14 (1.10, 1.17) ^a^	1.04 (1.03, 1.06) ^a^	0.98 (0.97, 0.99) ^a^	0.94 (0.93, 0.95) ^a^
	Type 2 Diabetes (reference: no diabetes)	1.31 (1.28, 1.35) ^a^	1.24 (1.22, 1.26) ^a^	1.11 (1.10, 1.12) ^a^	1.01 (1.01, 1.02) ^a^
	Microvascular/Macrovascular complications (reference: none)	**1.37 (1.28, 1.46)**	**1.31 (1.28, 1.34)**	**1.43 (1.41, 1.44)**	**1.61 (1.60, 1.63)**
Cognitive Disorder	Type 1 Diabetes (reference: no diabetes)	N/A	N/A	**0.89 (0.85, 0.94)**	**0.87 (0.85, 0.88)**
	Type 2 Diabetes (reference: no diabetes)	N/A	N/A	**0.96 (0.93, 0.99)**	**0.89 (0.88, 0.90)**
	Microvascular/Macrovascular complications (reference: none)	N/A	N/A	**2.45 (2.36, 2.54)**	**2.16 (2.13, 2.19)**
Chronic Pain	Type 1 Diabetes (reference: no diabetes)	1.06 (1.03, 1.09) ^a^	1.02 (1.01, 1.04) ^a^	0.94 (0.93, 0.95) ^a^	0.92 (0.91, 0.93) ^a^
	Type 2 Diabetes (reference: no diabetes)	1.44 (1.41, 1.48) ^a^	1.34 (1.33, 1.36) ^a^	1.12 (1.11, 1.13) ^a^	1.05 (1.04, 1.05) ^a^
	Microvascular/Macrovascular complications (reference: none)	**1.46 (1.37, 1.55)**	**1.35 (1.32, 1.38)**	**1.51 (1.50, 1.53)**	**1.77 (1.75, 1.78)**
Addiction	Type 1 Diabetes (reference: no diabetes)	1.03 (0.97, 1.09)	1.08 (1.06, 1.11) ^a^	0.83 (0.82, 0.84) ^a^	0.83 (0.81, 0.84) ^a^
	Type 2 Diabetes (reference: no diabetes)	**1.15 (1.09, 1.21)**	1.24 (1.22, 1.27) ^a^	0.99 (0.98, 1.00) ^a^	0.94 (0.93, 0.95) ^a^
	Microvascular/Macrovascular complications (reference: none)	**1.51 (1.36, 1.68)**	**1.33 (1.29, 1.37)**	**1.58 (1.56, 1.60)**	**1.58 (1.56, 1.60)**
Sleep Disorder	Type 1 Diabetes (reference: no diabetes)	0.97 (0.91, 1.04) ^a^	0.92 (0.90, 0.95) ^a^	0.93 (0.92, 0.94) ^a^	0.98 (0.97, 1.00) ^a^
	Type 2 Diabetes (reference: no diabetes)	1.54 (1.46, 1.63) ^a^	1.39 (1.36, 1.42) ^a^	1.25 (1.24, 1.26) ^a^	1.14 (1.12, 1.15) ^a^
	Microvascular/Macrovascular complications (reference: none)	**1.54 (1.38, 1.72)**	**1.41 (1.36, 1.46)**	**1.49 (1.48, 1.50)**	**1.52 (1.50, 1.53)**

OR, Odds ratio; CI, confidence interval; N/A, Not Applicable.

Bold represents a statistically significant (P-Value<0.05) difference in odds between individuals with type 1 diabetes and without diabetes, between individuals with type 2 diabetes and without diabetes, and between individuals with and without microvascular/macrovascular complications based on Wald Tests.

a Represents a statistically significant (P-Value<0.05) difference in odds between individuals with type 1 diabetes and individuals with type 2 diabetes based on Wald Tests.

### Cognitive Disorders

The results of the cognitive disorder models are presented in **Table 2**. After adjusting for the presence of microvascular/macrovascular complications, both individuals with type 1 diabetes (ages 40-60: OR 0.89, 95% CI: 0.85-0.94; ages 60+: OR 0.87, 95% CI: 0.85-0.88) and individuals with type 2 diabetes (ages 40-60: OR 0.96, 95% CI: 0.93-0.99; ages 60+: OR 0.89, 95% CI: 0.88-0.90) had significantly lower odds of having a cognitive disorder compared to those without diabetes. However, there was no significant difference between individuals with type 1 diabetes and individuals with type 2 diabetes. In all individuals, after adjusting for diabetes status, the presence of microvascular/macrovascular complications were independently associated with an increased odds of cognitive disorders (ages 40-60: OR 2.45, 95% CI: 2.36-2.54; ages 60+: OR 2.16, 95% CI: 2.13-2.19).

### Chronic Pain

The results of the chronic pain models are presented in **Table 2**. In all age strata and after adjusting for the presence of microvascular/macrovascular complications, individuals with type 2 diabetes (ages 0-20: OR 1.44, 95% CI: 1.41-1.48; ages 20-40: OR 1.34, 95% CI 1.33-1.36; ages 40-60: OR 1.12, 95% CI: 1.11-1.13; ages 60+: OR 1.05, 95% CI: 1.04-1.05) had significantly higher odds of chronic pain than both individuals with type 1 diabetes and those without diabetes. Compared to individuals without diabetes, individuals with type 1 diabetes aged 0-40 had significantly higher odds of chronic pain while individuals with type 1 diabetes aged 40+ had significantly lower odds of chronic pain than individuals with no diabetes (ages 0-20: OR 1.06, 95% CI: 1.03-1.09; ages 20-40: OR 1.02, 95% CI 1.01-1.04; ages 40-60: OR 0.94, 95% CI: 0.93-0.95; ages 60+: OR 0.92, 95% CI: 0.91-0.93). After adjusting for diabetes status, the presence of microvascular or macrovascular complications were independently associated with an increased odds of chronic pain amongst all age groups (ages 0-20: OR 1.46, 95% CI: 1.37-1.55; ages 20-40: OR 1.35, 95% CI 1.32-1.38; ages 40-60: OR 1.51, 95% CI: 1.50-1.53; ages 60+: OR 1.77, 95% CI: 1.75-1.78).

### Addiction Disorder

The results of the addiction disorder models are presented in **Table 2**. After adjusting for the presence of microvascular/macrovascular complications, only individuals with type 2 diabetes aged 0-40 had significant differences in the odds of addiction compared to individuals with type 1 diabetes and those without diabetes (ages 0-20: OR: 1.15, 95% CI 1.09-1.21; ages 20-40: OR: 1.24, 95% CI 1.22-1.27). Individuals aged 40+ with type 1 diabetes had a significantly smaller odds of addiction compared to individuals with type 2 diabetes and without diabetes (ages 40-60: OR 0.83, 95% CI: 0.82-0.84; ages 60+: OR 0.83, 95% CI: 0.81-0.84). In contrast, for individuals 0-20 years old, there were no differences in odds of addiction between individuals with type 1 diabetes and those without diabetes (OR 1.03, 95% CI 0.97-1.09), and individuals 20-40 years old experienced higher odds of addiction than individuals with no diabetes (OR 1.08, 95% CI 1.06-1.11). After adjusting for diabetes status, the presence of microvascular or macrovascular complications were independently associated with an increased odds of addiction (age 0-20: OR 1.51, 95% CI: 1.36-1.68; age 20-40: OR 1.33, 95% CI 1.29-1.37; age 40-60: OR 1.58, 95% CI: 1.56-1.60; age 60+: OR 1.58, 95% CI: 1.56-1.60).

### Sleep Disorders

The results of the sleep disorder models are presented in **Table 2**. In all age groups and after adjusting for the presence of microvascular/macrovascular complications, individuals with type 2 diabetes had significantly higher odds of having sleep disorders compared to individuals without diabetes, (ages 0-20: OR 1.54, 95% CI: 1.46-1.63; ages 20-40: OR 1.39, 95% CI 1.36-1.42; ages 40-60: OR 1.25, 95% CI: 1.24-1.26; ages 60+: OR 1.14, 95% CI: 1.12-1.15). Individuals with type 1 diabetes ages 20-60 had significantly lower odds of sleep disorders than individuals without diabetes (ages 20-40: OR 0.92, 95% CI: 0.90-0.95; ages 40-60: OR 0.93, 95% CI: 0.92-0.94). After adjusting for diabetes status, the presence of microvascular or macrovascular complications was independently associated with an increased odds of sleep disorders across age strata (ages 0-20: OR 1.54, 95% CI: 1.38-1.72; ages 20-40: OR 1.41, 95% CI 1.36-1.46; ages 40-60: OR 1.49, 95% CI: 1.48-1.50; ages 60+: OR 1.52, 95% CI: 1.50-1.53).

### Effect of Specific Microvascular and Macrovascular Complications

When separating the effects of complications into microvascular, macrovascular, and amputations, nearly all effects remained positive and statistically significant, as detailed in **Supplementary Table S2**. Macrovascular complications had the largest effect size for 14 out of 18 comparisons. The models with individual complication effects, detailed in **Supplementary Table S3**, revealed that amongst microvascular complications, neuropathy had the largest effect size for 17 out of 18 comparisons. Amongst macrovascular complications, stroke had the largest effect size for 12 out of 18 comparisons.

## Discussion

To our knowledge, this is the largest US study to examine the prevalence of neuropsychological outcomes among a nationally representative population of privately insured individuals with type 1 and type 2 diabetes and controls without diabetes. Furthermore, we are unaware of studies that have evaluated the independent effects of diabetes status and microvascular/macrovascular complications on these neuropsychological outcomes. We found that the prevalence of neuropsychological outcomes (mental health, chronic pain, addiction, and sleep disorders) was higher in individuals with type 2 diabetes compared to type 1 diabetes, and in individuals with type 1 diabetes compared to those without diabetes. For cognitive disorders, microvascular complications, and macrovascular complications, the prevalence was highest in those with type 1 diabetes, followed by those with type 2 diabetes and then those without diabetes. Microvascular and macrovascular complications were consistently associated with higher odds for all five neuropsychological outcomes, independent of diabetes status. Interestingly, after adjusting for the presence of microvascular and macrovascular complications, individuals with type 1 diabetes had similar odds of developing neuropsychological outcomes compared to those without diabetes (no odds ratios >1.15). In contrast, individuals with type 2 diabetes are more likely to experience mental health, chronic pain, and sleep disorders even after adjusting for microvascular and macrovascular complications.

Despite a higher prevalence of neuropsychological outcomes, we found that individuals with type 1 diabetes had similar or reduced odds of developing all neuropsychological outcomes compared to individuals without diabetes, after adjusting for the presence of microvascular and macrovascular complications. Thus, microvascular and macrovascular complications likely play a fundamental role in the development of neuropsychological outcomes in individuals with type 1 diabetes. One explanation of our results is that the same pathophysiologic processes that drive microvascular and macrovascular complications also drive neuropsychological outcomes. For instance, individuals with a longer duration of diabetes or worse glycemic control are more likely to develop complications, and these same factors may also increase the risk of neuropsychological outcomes. Unfortunately, our database does not contain information on diabetes duration or severity to address this important question. Another possibility is that the complications themselves lead to worse neuropsychological outcomes, either directly through downstream consequences that result from these complications or indirectly through reduced quality of life and disease burden. A combination of these two explanations is likely and should be the focus of future studies. In addition, future studies should focus on the role of neuropathy and stroke as these were the individual microvascular/macrovascular complications that resulted in the highest odds of neuropsychological outcomes. Furthermore, since microvascular and macrovascular complications are more common in individuals with type 1 diabetes and are a major driver of the higher prevalence of neuropsychological outcomes in these individuals, our results highlight the importance of preventing these complications.

In contrast, after adjusting for the presence of microvascular and macrovascular complications, individuals with type 2 diabetes were still at higher risk for developing three neuropsychological outcomes: mental health disorders, chronic pain, and sleep disorders, compared to both individuals with type 1 diabetes, and individuals without diabetes. These results indicate that factors beyond microvascular and macrovascular complications likely contribute to the development of these wide-ranging neuropsychological conditions in individuals with type 2 diabetes. Since individuals with type 2 diabetes have a higher prevalence of metabolic risk factors than individuals with type 1 diabetes and the general population, these other metabolic factors may contribute to the higher prevalence of neuropsychological outcomes.

Supporting this hypothesis, metabolic risk factors other than hyperglycemia have been shown to be associated with multiple neuropsychological outcomes. Specifically, meta-analyses demonstrated associations between obesity, metabolic control and mental health disorders such as anxiety and depression (24, 25). Similarly, a meta-analysis revealed associations between overweight and obesity with chronic pain (26). Moreover, obesity also increases the likelihood of lower quality sleep and sleep apnea (27). Given the robust literature linking obesity and other metabolic risk factors with neuropsychological outcomes and the high prevalence of these comorbidities with type 2 diabetes, the higher prevalence of neuropsychological outcomes in the type 2 compared to the type 1 diabetes population is at least partially explained.

Another possibility is that individuals that have or are susceptible to neuropsychological outcomes may be more likely to develop type 2 diabetes. Though the majority of the literature focuses on risks in individuals that already have type 2 diabetes, a systematic review (28) found that depressed adults have a 37% increased risk of developing type 2 diabetes. While demographic factors are also different between type 1 and type 2 diabetes populations, our comparisons are adjusted for many key factors including age, sex, race, ethnicity, and socioeconomic status. Given that microvascular and macrovascular complications are not the sole driving force behind neuropsychological outcomes in individuals with type 2 diabetes, studies are needed to determine the other key risk factors including demographic factors.

Individuals that experienced any microvascular complications, macrovascular complications or amputations had higher odds of having each neuropsychological outcome, suggesting that these complications are the primary driver for a wide range of neuropsychological outcomes, regardless of diabetes status. Although macrovascular complications were less prevalent than microvascular complications, macrovascular complications were associated with a higher odds of neuropsychological outcomes compared to microvascular complications in 14 out of the 18 models we evaluated. The macrovascular and microvascular complications having the largest associations with neuropsychological outcomes were stroke (12 out of 18 comparisons) and neuropathy (17 out of 18 comparisons) respectively. These results are congruent with previous studies that have found that dementia, mental health disorders, chronic pain, and sleep disorders were common in individuals following a stroke (29–33). In addition, neuropathy has been previously linked to chronic pain, various mental health disorders, sleep disorders, lower cognitive performance, and inhalant addiction (34–39). Given that individuals with these complications have a higher risk for these neuropsychological outcomes, preventing or improving complications such as neuropathy or stroke in individuals with diabetes may simultaneously improve their neuropsychological prospects, and therefore, should be the focus of future studies.

Limitations of the current study include possible disease misclassification using ICD-9/ICD-10 codes. However, many of our definitions have been validated with high positive predictive values. Separately, claims data lack the necessary detailed clinical information to assess the severity of microvascular/macrovascular complications, neuropsychological conditions, and diabetes. In addition, our analyses may have differentially captured severe neuropsychological outcomes, as only such cases would prompt a visit to a provider and result in a diagnostic code. Furthermore, the generalizability to other populations such as those that are not privately insured is unclear. On the other hand, the large-scale claims data allowed us to identify a wide range of neuropsychological outcomes across many age ranges, including older populations with type 1 diabetes.

In summary, individuals with type 1 diabetes have a higher prevalence of neuropsychological outcomes compared to those without diabetes. However, after adjusting for the presence of microvascular or macrovascular complications, type 1 diabetes was not associated with an increased odds of neuropsychological outcomes compared to individuals without diabetes. Furthermore, microvascular and macrovascular complications are independently associated with neuropsychological outcomes. Specifically, we identified stroke and neuropathy as major risk factors for most neuropsychological outcomes. Therefore, prevention of microvascular and macrovascular complications will likely reduce neuropsychological outcomes either as the result of similar pathophysiologic processes or by preventing the direct and indirect consequences of these complications. In contrast, individuals with type 2 diabetes were at increased odds of multiple neuropsychological outcomes compared to those with type 1 diabetes, even after adjusting for presence of microvascular/macrovascular complications. This indicates that in individuals with type 2 diabetes, other factors (such as obesity) may lead to neuropsychological complications. Alternatively, it is possible that neuropsychological complications may result in type 2 diabetes onset.

## Data Availability Statement

The data analyzed in this study is subject to the following licenses/restrictions: BC is the guarantor of this work and, as such, had full access to all the data in the study and takes responsibility for the integrity of the data and the accuracy of the data analysis. Requests to access these datasets should be directed to bcallagh@med.umich.edu.

## Ethics Statement

The studies involving human participants were reviewed and approved by Institutional Review Board of the University of Michigan (HUM00176199). Written informed consent from the participants’ legal guardian/next of kin was not required to participate in this study in accordance with the national legislation and the institutional requirements.

## Author Contributions

NP was involved in the data management, study design, statistical analysis, interpretation of data, and wrote the manuscript. ER was involved in the study design, interpretation of the statistical analysis, and critical revisions of the manuscript. MB was involved in the study design, interpretation of the data, and critical revisions of the manuscript. KM-S was involved in data interpretation and manuscript revision. DA was involved in data interpretation and manuscript revision. JL was involved in data interpretation and the critical revisions of the manuscript. RP-B was involved in data interpretation and the critical revisions of the manuscript. EF was involved in the interpretation of the statistical analysis and critical revisions of the manuscript. BC was involved in the study design, interpretation of the statistical analysis and critical revisions of the manuscript. All authors contributed to the article and approved the submitted version.

## Funding

This work was funded by the Juvenile Diabetes Research Foundation. NP is supported by the JDRF. ER is supported by NIH T32NS0007222. KM-S is supported by the JDRF. DA is supported by the JDRF and M-Diabetes Center of Excellence through the Psychological and Cognitive Impacts of Type 1 Diabetes project (10/2019 – 9/2024) as well as NICHHD (5UH3HD087979-05) through the Target Self-regulation to Promote Adherence and Health Behaviors in Children project (9/2015 – 8/2021). JL is supported by the Elizabeth Weiser Caswell Diabetes Institute at the University of Michigan; research grants G-1903-144168 from the Michigan Health Endowment Fund; 5-COE-2019-861-S-B from JDRF; N030009 from the Gerber Foundation; P30 Grant12959224, UH3HD087979, and UH3HD087979-04S1 from the National Institutes of Health; Helmsley Charitable Trust; and University of Michigan MCubed. RP-B is supported by NIH/NIDDK-1-R01-DK-107956-01; NIH U01DK119083, NIDDK/NHLBI 1UG3AT009150-01, and JDRF Grant 5-COE-2019-861-S-B. EF is supported by NIH R24DK082841, NIH R21NS102924, The NeuroNetwork for Emerging Therapies, The Robert and Katherine Jacobs Environmental Health Initiative, The Robert E. Nederlander Sr. Program for Alzheimer’s Research, The Sinai Medical Staff Foundation, the Milstein Family Foundation. BC is supported by NIH R01DK115687 and the JDRF.

## Conflict of Interest

JL is on the medical advisory board for GoodRx. RP-B consults for Novo Nordisk, Boehringer Ingelheim, Regenacy, Averitas and Nevro. EF consults for Novartis. BC consults for a PCORI grant, DynaMed, receives research support from the American Academy of Neurology and performs medical legal consultations including consultations for the Vaccine Injury Compensation Program.

The remaining authors declare that the research was conducted in the absence of any commercial or financial relationships that could be construed as a potential conflict of interest.

The reviewer [SB] declared a shared affiliation with the authors to the handling editor at time of review.

## Publisher’s Note

All claims expressed in this article are solely those of the authors and do not necessarily represent those of their affiliated organizations, or those of the publisher, the editors and the reviewers. Any product that may be evaluated in this article, or claim that may be made by its manufacturer, is not guaranteed or endorsed by the publisher.

## Abbreviations

ICD-9/ICD-10, International Classification of Diseases, 9^th,^ and 10^th^ editions respectively.

## References

[1] Harding JL Pavkov ME Magliano DJ Shaw JE Gregg EW. Global Trends in Diabetes Complications: A Review of Current Evidence. Diabetologia (2019) 62(1):3–16. doi: 10.1007/s00125-018-4711-2 30171279

[2] Hahl J Hämäläinen H Sintonen H Simell T Arinen S Simell O. Health-Related Quality of Life in Type 1 Diabetes Without or With Symptoms of Long-Term Complications. Qual Life Res (2002) 11(5):427–36. doi: 10.1023/A:1015684100227 12113390

[3] Roy T Lloyd CE. Epidemiology of Depression and Diabetes: A Systematic Review. J Affect Disord (2012) 142:S8–S21. doi: 10.1016/S0165-0327(12)70004-6 23062861

[4] Li W Huang E Gao S. Type 1 Diabetes Mellitus and Cognitive Impairments: A Systematic Review. J Alzheimers Dis (2017) 57(1):29–36. doi: 10.3233/JAD-161250 28222533

[5] Tran ST Salamon KS Hainsworth KR Kichler JC Davies WH Alemzadeh R. Pain Reports in Children and Adolescents With Type 1 Diabetes Mellitus. J Child Health Care (2015) 19(1):43–52. doi: 10.1177/1367493513496908 23939723

[6] Molvær AK Iversen MM Igland J Peyrot M Tell GS Holte KB. Higher Levels of Bodily Pain in People With Long-Term Type 1 Diabetes: Associations With Quality of Life, Depressive Symptoms, Fatigue and Glycaemic Control – the Dialong Study. Diabetes Med (2020) 37(9):1569–77. doi: 10.1111/dme.14331 32446279

[7] Pastor A Conn J Teng J O'Brien CL Loh M Collins L. Alcohol and Recreational Drug Use in Young Adults With Type 1 Diabetes. Diabetes Res Clin Pract (2017) 130:186–95. doi: 10.1016/j.diabres.2017.05.026 28646702

[8] Reutrakul S Thakkinstian A Anothaisintawee T Chontong S Borel A-L Perfect MM. Sleep Characteristics in Type 1 Diabetes and Associations With Glycemic Control: Systematic Review and Meta-Analysis. Sleep Med (2016) 23:26–45. doi: 10.1016/j.sleep.2016.03.019 27692274 PMC9554893

[9] Klompas M Eggleston E McVetta J Lazarus R Li L Platt R. Automated Detection and Classification of Type 1 Versus Type 2 Diabetes Using Electronic Health Record Data. Diabetes Care (2013) 36(4):914–21. doi: 10.2337/dc12-0964 PMC3609529 23193215

[10] Sørensen MJ Mors O Thomsen PH. DSM-IV or ICD-10-DCR Diagnoses in Child and Adolescent Psychiatry: Does It Matter? Eur Child Adolesc Psychiatry (2005) 14(6):335–40. doi: 10.1007/s00787-005-0482-7 16220218

[11] Fiest KM Jette N Quan H Germaine-Smith C Metcalfe A Patten SB. Systematic Review and Assessment of Validated Case Definitions for Depression in Administrative Data. BMC Psychiatry (2014) 14(1):289. doi: 10.1186/s12888-014-0289-5 25322690 PMC4201696

[12] Fernández A Rubio-Valera M Bellón JA Pinto-Meza A Luciano JV Mendive JM. Recognition of Anxiety Disorders by the General Practitioner: Results From the DASMAP Study. Gen Hosp Psychiatry (2012) 34(3):227–33. doi: 10.1016/j.genhosppsych.2012.01.012 22341732

[13] Wilchesky M Tamblyn RM Huang A. Validation of Diagnostic Codes Within Medical Services Claims. J Clin Epidemiol (2004) 57(2):131–41. doi: 10.1016/S0895-4356(03)00246-4 15125622

[14] Januel JM Luthi JC Quan H Borst F Taffé P Ghaliet WA. Improved Accuracy of Co-Morbidity Coding Over Time After the Introduction of ICD-10 Administrative Data. BMC Health Serv Res (2011) 11(1):194. doi: 10.1186/1472-6963-11-194 21849089 PMC3170597

[15] Rizzuto D Feldman AL Karlsson IK Dahl Aslan AK Gatz M Pedersen NL. Detection of Dementia Cases in Two Swedish Health Registers: A Validation Study. J Alzheimers Dis (2018) 61(4):1301–10. doi: 10.3233/JAD-170572 PMC6218116 29376854

[16] Kim HM Smith EG Stano CM Ganoczy D Zivin K Walters H. Validation of Key Behaviourally Based Mental Health Diagnoses in Administrative Data: Suicide Attempt, Alcohol Abuse, Illicit Drug Abuse and Tobacco Use. BMC Health Serv Res (2012) 12(1):18. doi: 10.1186/1472-6963-12-18 22270080 PMC3280157

[17] Davis J Robinson R Le X. Incidence and Impact of Pain Conditions and Comorbid Illnesses. J Pain Res (2011) 4:331–45. doi: 10.2147/JPR.S24170 PMC3215513 22090802

[18] Jolley RJ Liang Z Peng M Pendharkar SR Tsai W Chen G. Identifying Cases of Sleep Disorders Through International Classification of Diseases (ICD) Codes in Administrative Data. Int J Popul Data Sci (2018) 3(1):448. doi: 10.23889/ijpds.v3i1.448 32935008 PMC7299484

[19] Callaghan BC Reynolds E Banerjee M Kerber KA Skolarus LE Burke JF. Longitudinal Pattern of Pain Medication Utilization in Peripheral Neuropathy Patients. PAIN (2019) 160(3):592–9. doi: 10.1097/j.pain.0000000000001439 PMC6377284 30418352

[20] Newton KM Wagner EH Ramsey SD McCulloch D Evans R Sandhu N. The Use of Automated Data to Identify Complications and Comorbidities of Diabetes. J Clin Epidemiol (1999) 52(3):199–207. doi: 10.1016/S0895-4356(98)00161-9 10210237

[21] Jones SA Gottesman RF Shahar E Wruck L Rosamond WD. Validity of Hospital Discharge Diagnosis Codes for Stroke: The Atherosclerosis Risk in Communities Study. Stroke (2014) 45(11):3219–25. doi: 10.1161/STROKEAHA.114.006316 PMC4290877 25190443

[22] Wang Y Cai H Li C Jiang Z Wang L Song J. Optimal Caliper Width for Propensity Score Matching of Three Treatment Groups: A Monte Carlo Study. Hills RK, Ed. PloS One (2013) 8(12):e81045. doi: 10.1371/journal.pone.0081045 24349029 PMC3859481

[23] Quan H Sundararajan V Halfon P Fong A Burnand B Luthi J-C. Coding Algorithms for Defining Comorbidities in ICD-9-CM and ICD-10 Administrative Data. Med Care (2005) 43(11):1130–9. doi: 10.1097/01.mlr.0000182534.19832.83 16224307

[24] Lustman PJ Anderson RJ Freedland KE de Groot M Carney RM Clouse RE. Depression and Poor Glycemic Control: A Meta-Analytic Review of the Literature. Diabetes Care (2000) 23(7):934–42. doi: 10.2337/diacare.23.7.934 10895843

[25] Sutaria S Devakumar D Yasuda SS Das S Saxena S. Is Obesity Associated With Depression in Children? Systematic Review and Meta-Analysis. Arch Dis Child (2019) 104(1):64–74. doi: 10.1136/archdischild-2017-314608 29959128

[26] Qian M Shi Y Yu M. The Association Between Obesity and Chronic Pain Among Community-Dwelling Older Adults: A Systematic Review and Meta-Analysis. Geriatr Nurs (Lond) (2021) 42(1):8–15. doi: 10.1016/j.gerinurse.2020.10.017 33197704

[27] Cappuccio FP Taggart FM Kandala NB Currie A Peile E Stranges S. Meta-Analysis of Short Sleep Duration and Obesity in Children and Adults. Sleep (2008) 31(5):619–26. doi: 10.1093/sleep/31.5.619 PMC2398753 18517032

[28] Knol MJ Twisk JWR Beekman ATF Heine RJ Snoek FJ Pouwer F. Depression as a Risk Factor for the Onset of Type 2 Diabetes Mellitus. A Meta-Analysis. Diabetologia (2006) 49(5):837–45. doi: 10.1007/s00125-006-0159-x 16520921

[29] Craig L Hoo ZL Yan TZ Wardlaw J Quinn TJ. Prevalence of Dementia in Ischaemic or Mixed Stroke Populations: Systematic Review and Meta-Analysis. J Neurol Neurosurg Psychiatry (2022) 93(2):180–7. doi: 10.1136/jnnp-2020-325796 PMC8784999 34782389

[30] Naghavi FS Koffman EE Lin B Du J. Post-Stroke Neuronal Circuits and Mental Illnesses. Int J Physiol Pathophysiol Pharmacol (2019) 11(1):1–11. 30911356 PMC6420715

[31] Robinson RG Jorge RE. Post-Stroke Depression: A Review. Am J Psychiatry (2016) 173(3):221–31. doi: 10.1176/appi.ajp.2015.15030363 26684921

[32] Harrison RA Field TS. Post Stroke Pain: Identification, Assessment, and Therapy. Cerebrovasc Dis (2015) 39(3-4):190–201. doi: 10.1159/000375397 25766121

[33] Khot SP Morgenstern LB. Sleep and Stroke. Stroke (2019) 50(6):1612–7. doi: 10.1161/STROKEAHA.118.023553 PMC6640639 31043150

[34] Baron R Binder A Wasner G. Neuropathic Pain: Diagnosis, Pathophysiological Mechanisms, and Treatment. Lancet Neurol (2010) 9(8):807–19. doi: 10.1016/S1474-4422(10)70143-5 20650402

[35] Naranjo C Del Reguero L Moratalla G Hercberg M Valenzuela M Failde I. Anxiety, Depression and Sleep Disorders in Patients With Diabetic Neuropathic Pain: A Systematic Review. Expert Rev Neurother (2019) 19(12):1201–9. doi: 10.1080/14737175.2019.1653760 31393191

[36] Naranjo C Ortega-Jiménez P del Reguero L Moratalla G Failde I. Relationship Between Diabetic Neuropathic Pain and Comorbidity. Their Impact on Pain Intensity, Diabetes Complications and Quality of Life in Patients With Type-2 Diabetes Mellitus. Diabetes Res Clin Pract (2020) 165:108236. doi: 10.1016/j.diabres.2020.108236 32470476

[37] Lin YJ Kao TW Chen WL. Relationship Between Peripheral Neuropathy and Cognitive Performance in the Elderly Population. Med (Baltimore) (2021) 100(20):e26071. doi: 10.1097/MD.0000000000026071 PMC8137106 34011128

[38] Winstock AR Ferris JA. Nitrous Oxide Causes Peripheral Neuropathy in a Dose Dependent Manner Among Recreational Users. J Psychopharmacol (Oxf) (2020) 34(2):229–36. doi: 10.1177/0269881119882532 31679459

[39] Staff NP. Peripheral Neuropathies Due to Vitamin and Mineral Deficiencies, Toxins, and Medications. Continuum Lifelong Learn Neurol (2020) 26(5):1280–98. doi: 10.1212/CON.0000000000000908 33003002

