# Supplementary material for: Neuropsychological Outcomes in Individuals With Type 1 and Type 2 Diabetes
Source: Front Endocrinol (Lausanne). 2022 Mar 4;13:834978. doi: 10.3389/fendo.2022.834978 (PMC8934404; doi:10.3389/fendo.2022.834978)
Supplement: Supplementary file 1 [file DataSheet_1.docx]

Supplementary Table S1: ICD-9/10 codes

| **Condition** | **ICD-9** | | **ICD-10** |
| --- | --- | --- | --- |
| **Diabetes** |  | |  |
| Type 1 Diabetes | 250.x1, 250.x3 | | E10.xx |
| Type 2 Diabetes | 250.x0, 250.x2 | | E11.xx |
| **Microvascular Complications** |  | |  |
| Retinopathy | 362.0, 362.01–362.06, 362.10 | | E11.31x-E11.35x, E10.31x-E10.35x,E08.31x-E08.35x, E09.31x-E09.35x,E13.31x-E13.35x |
| Neuropathy | 356.x, 357.1-8, 357.82, 357.89, and 357.9 | | G60.x, G62.x, G63.x, G65.2, E08.42, E09.42, E10.42, E11.42, E13.42 |
| Nephropathy | 250.4x, 585.x | | E08.2x, E09.2x,E10.2x,E11.2x,E13.2x, N18.x |
| **Macrovascular Complications** |  | |  |
| Myocardial infarction | 410.x | | I21.x |
| Stroke | 433.x1, 434.x1 | | I63.x |
| Peripheral Vascular Disease | 440.2-4, 440.9, 443.8, 443.89, 443.9, 250.7x | | I70.2x-7x, I70.91-92, I73.x, I73.8, I73.89, I73.9, E08.5x,E09.5x,E10.5x,E11.5x,E13.5x |
| **Other Complications** |  | |  |
| Amputations | 84.11-84.16, 84.17, 28800, 28805, 28810, 28820, 28825 | |  |
| **Mental Health** | |  |  |
| Anxiety | | 300.0x | F41.x |
| Depression | | 296.2x, 296.3x, 311.x, 300.4 | F32.x, F33.x, F34.x, F39.x |
| Post-Traumatic Stress Disorder | | 309.81 | F43.1 |
| Eating disorders | | 307.1, 307.5x | F50.x |
| Adjustment disorder | | 309.x, except 309.81 | F43.x EXCEPT 43.1 |
| Dissociative, conversion disorder | | 300.1x, 300.6-8x | F44.x-45.x |
| Behavioral and emotional disorders with onset usually in childhood/adolescence | | 313.x, 314.x | F90.x-98.x |
| Attention Deficit Hyperactivity Disorder | | 314.0x | F90.x |
| **Cognitive** |  | |  |
| Dementia | 290.0-4, 294.1-2, 331.0-2, 331.7, 331.8, 331.92, 331.89, 331.9 | | G30.x, G31.0x, G31.1, G31.8, G31.83, G31.89, G31.9, G32, G32.8, G32.89, F01.x-F03.x |
| Mild Cognitive Impairment | 331.83 | | G31.84 |
| Alzheimer’s | 331.0 | | G30.x |
| Vascular dementia | 290.4x | | F01.x |
| **Chronic Pain** |  | |  |
| Chronic Pain | 729.1, 729.0, 346.xx, 339.1x, 339.02, 339.04, 307.81, 564.1x, 306.4, 780.71, 780.79, 524.6x, 617.xx, 625.7x, 595.1, 595.2, 595.9, 724.2, 724.5, 053.10, 053.11, 053.12, 053.13, 053.14, 053.19, 350.1, (042, 079.53, 795.71, V08) AND (338.29, 338.4, 780.96), (430–434.9, 436–437.9) AND 338.0, 724.4, 337.2, 952 AND (338.29, 338.4, 780.96), 338.18, 338.28, 353.6, 723.4, (340 AND 338.0), 729.1715.0, 715.1, 715.2, 715.8, 715.9, 721.0, 721.1, 721.2, 721.3, 721.41, 721.42, 722.83, 724.02, 724.2, 724.5, 724.8, 346.0, 346.1, 346.2, 346.8, 346.9, 714.0, 714.1, 714.2, 714.30, 714.31, 714.32, 714.33, 714.4, 714.81, 720.0, 720.1, 720.2, 720.81, 720.89, 696, 338.3, 564.1, 788.41 AND (625.8 OR 625.9), 595.1 | | M97.7, M79.0, G43.xx, G44.2xx, G44.029, G44.049, K58.1, K58.2, K58.8, K58.9, F45.8, R53.82, G93.3, R53.1, R53.81, R53.83, M26.601, M26.602, M26.603, M26.609, M26.69, M26.611, M26.612, M26.613, M26.619, M26.621, M26.622, M26.623, M26.629, M26.631, M26.632, M26.633, M26.639, M26.69, N80.xx, N94.81x, N30.10, N30.11, N30.20, N30.21, N30.90, N30.91, M54.5, M54.89, M54.9, B02.29, B02.21, B02.22, B02.23, B02.24, B02.29, G50.0, (B20, B97.35, R75, Z21) AND (G89.29, G89.4, R52), ((I60-I62, I65-I66, I67.xx) AND G89.0), M54.14, M54.15, M54.16, M54.17, G90.59, G90.519, G90.529, ((S34.109A, S34.139A, S34.3XXA, S14.109A, S24.109A, S34.109A, S34.139A) AND (G89.29, G89.4, R52)), G89.18, G89.28, G54.6, G54.7, M54.12, M54.13, (G35 AND G89.0), M60.9, M79.1, M79.7, M15.0, M15.9, M15.1, M15.2, M19.91, M19.019, M19.029, M19.039, M19.049, M16.10, M17.10, M19.079, M19.91, M19.93, M19.219, M19.229, M19.239, M19.249, M16.7, M17.5, M19.279, M19.93, M15.3, M15.8, M15.9, M19.90, M18.9, M16.9, M17.9, M47.812, M47.12, M47.814, M47.817, M47.14, M47.16, M96.1, M48.061, M54.5, M54.89, M54.9, M54.08, G43.109, G43.119, G43.101, G43.111, G43.009 G43.019, G43.001, G43.011, G43.809, G43A0, G43.B0, G43.C0, G43.D0, G43.819, G43.A1, G43.B1, G43.C1, G43.D1, G43.801, G43.811, G43.809, G43.801, G43.811, G43.909, G43.919, G43.901, G43.911, M06.9, M05.00, M05.30, M05.60, M06.1, M08.00, M08.3, M08.40, M12.00, M05.10, M45.9, M46.00, M46.1, M49.80, M46.80, L40.54, L40.59, L40.0, L40.1, L40.2, L40.3, L40.4, L40.8, L41.0, L41.1, L41.8, L42, L44.0, L30.5, L44.8, G89.3, K58.1, K58.2, K58.8, K58.9, R35.0 AND (N84.89 OR N94.89), N30.10, N30.11 |
| **Addiction** |  | |  |
| Alcoholism | 303.x, 305.0x, 291.x | | F10.x |
| Opioids | 304.00-304.03 | | F11.x |
| Cannabis | 304.30-304.33 | | F12.x |
| Sedatives | 304.10-304.13 | | F13.x |
| Cocaine | 304.20-304.23 | | F14.x |
| Other stimulant | 304.40-304.43 | | F15.x |
| Hallucinogen | 304.50-304.53 | | F16.x |
| Nicotine | 305.1 | | F17.x |
| Inhalant | NA | | F18.x |
| Other psychoactive and non-psychoactive | 304.9, 304.6 | | F19.x, F55.x |
| **Sleep** |  | |  |
| Sleep disorders | 307.4x, 327.x, 347.x, 780.5x | | G47.x and F51.x |
| Insomnia | 327.0x, 307.41-44, 780.51, 780.52 | | G47.0x and F51.0x |
| Hypersomnia | 327.1x, 780.53, 780.54 | | G47.1x and F51.1x |
| Obstructive Sleep Apnea | 327.23 | | G47.33 |
| Circadian rhythm | 327.3x, 307.45 | | G47.2, F51.8 |

Supplementary Table S2: The association between diabetes status and microvascular complications, macrovascular complications, and neuropsychological outcomes stratified by age

| **Model Outcome** | **Covariate** | **Ages 0-20**  **OR (95% CI)** | **Ages 20-40**  **OR (95% CI)** | **Ages 40-60**  **OR (95% CI)** | **Ages 60+**  **OR (95% CI)** |
| --- | --- | --- | --- | --- | --- |
| Mental Health Disorder | Type 1 Diabetes (reference: no diabetes) | **1.14 (1.10, 1.17) ^a^** | **1.05 (1.03, 1.06) ^a^** | **0.98 (0.97, 0.99) ^a^** | **0.94 (0.93, 0.95) ^a^** |
|  | Type 2 Diabetes (reference: no diabetes) | **1.31 (1.28, 1.35) ^a^** | **1.24 (1.22, 1.26) ^a^** | **1.11 (1.10, 1.12) ^a^** | **1.01 (1.00, 1.02) ^a^** |
|  | Microvascular complications (reference: none) | **1.34 (1.25, 1.44)** | **1.24 (1.21, 1.27)** | **1.32 (1.31, 1.33)** | **1.33 (1.31, 1.34)** |
|  | Macrovascular complications (reference: none) | **1.37 (1.18, 1.58)** | **1.37 (1.30, 1.44)** | **1.36 (1.34, 1.38)** | **1.46 (1.44, 1.47)** |
|  | Amputation (reference: none) | **1.42 (1.14, 1.78)** | **1.18 (1.09, 1.27)** | **1.14 (1.11, 1.17)** | **1.21 (1.18, 1.23)** |
| Cognitive Disorder | Type 1 Diabetes (reference: no diabetes) | NA | NA | **0.86 (0.81, 0.90) ^a^** | **0.87 (0.85, 0.88) ^a^** |
|  | Type 2 Diabetes (reference: no diabetes) | NA | NA | **0.95 (0.91, 0.98) ^a^** | **0.90 (0.89, 0.91) ^a^** |
|  | Microvascular complications (reference: none) | NA | NA | **1.72 (1.65, 1.79)** | **1.35 (1.33, 1.36)** |
|  | Macrovascular complications (reference: none) | NA | NA | **2.17 (2.08, 2.26)** | **1.98 (1.95, 2.00)** |
|  | Amputation (reference: none) | NA | NA | **1.17 (1.09, 1.27)** | **1.30 (1.27, 1.32)** |
| Chronic Pain | Type 1 Diabetes (reference: no diabetes) | **1.06 (1.03, 1.09) ^a^** | **1.03 (1.01, 1.04) ^a^** | **0.94 (0.93, 0.95) ^a^** | **0.92 (0.91, 0.93) ^a^** |
|  | Type 2 Diabetes (reference: no diabetes) | **1.44 (1.41, 1.48) ^a^** | **1.34 (1.33, 1.36) ^a^** | **1.12 (1.11, 1.13) ^a^** | **1.04 (1.04, 1.05) ^a^** |
|  | Microvascular complications (reference: none) | **1.40 (1.31, 1.50)** | **1.27 (1.24, 1.29)** | **1.36 (1.35, 1.37)** | **1.48 (1.47, 1.49)** |
|  | Macrovascular complications (reference: none) | **1.62 (1.40, 1.88)** | **1.57 (1.49, 1.65)** | **1.53 (1.51, 1.55)** | **1.60 (1.59, 1.62)** |
|  | Amputation (reference: none) | **1.25 (1.01, 1.55)** | **1.19 (1.11, 1.28)** | **1.16 (1.13, 1.19)** | **1.28 (1.25, 1.31)** |
| Addiction | Type 1 Diabetes (reference: no diabetes) | 1.03 (0.97, 1.09) | **1.09 (1.06, 1.12) ^a^** | **0.85 (0.84, 0.86) ^a^** | **0.83 (0.82, 0.85) ^a^** |
|  | Type 2 Diabetes (reference: no diabetes) | **1.15 (1.09, 1.21)** | **1.24 (1.22, 1.27) ^a^** | 1.00 (0.99, 1.01) **^a^** | **0.95 (0.93, 0.96) ^a^** |
|  | Microvascular complications (reference: none) | **1.49 (1.33, 1.67)** | **1.22 (1.18, 1.27)** | **1.25 (1.23, 1.27)** | **1.21 (1.19, 1.23)** |
|  | Macrovascular complications (reference: none) | **1.30 (1.02, 1.66)** | **1.36 (1.27, 1.45)** | **1.67 (1.65, 1.70)** | **1.53 (1.51, 1.55)** |
|  | Amputation (reference: none) | **1.51 (1.10, 2.08)** | **1.36 (1.23, 1.50)** | **1.14 (1.11, 1.17)** | **1.07 (1.04, 1.10)** |
| Sleep Disorder | Type 1 Diabetes (reference: no diabetes) | 0.97 (0.91, 1.04) **^a^** | **0.92 (0.90, 0.95) ^a^** | **0.92 (0.91, 0.94) ^a^** | **0.97 (0.96, 0.99) ^a^** |
|  | Type 2 Diabetes (reference: no diabetes) | **1.54 (1.46, 1.63) ^a^** | **1.39 (1.36, 1.42) ^a^** | **1.25 (1.23, 1.26) ^a^** | **1.13 (1.12, 1.14) ^a^** |
|  | Microvascular complications (reference: none) | **1.44 (1.27, 1.63)** | **1.31 (1.26, 1.35)** | **1.38 (1.36, 1.39)** | **1.39 (1.37, 1.40)** |
|  | Macrovascular complications (reference: none) | **1.83 (1.43, 2.33)** | **1.52 (1.42, 1.62)** | **1.37 (1.35, 1.39)** | **1.28 (1.27, 1.30)** |
|  | Amputation (reference: none) | 1.40 (0.99, 2.00) | **1.23 (1.11, 1.36)** | **1.10 (1.07, 1.13)** | **1.09 (1.06, 1.11)** |

OR= Odds ratio, CI= confidence interval

**Bold Confidence intervals show a statistically significant different from their reference group**

^a^ represents a significant difference in odds between individuals with type 1 diabetes and individuals with type 2 diabetes

Supplementary Table S3: The association between diabetes status and seven individual complications and neuropsychological outcomes stratified by age

| **Model Outcome** | **Covariate** | **Ages 0-20**  **OR (95% CI)** | **Ages 20-40**  **OR (95% CI)** | **Ages 40-60**  **OR (95% CI)** | **Ages 60+**  **OR (95% CI)** |
| --- | --- | --- | --- | --- | --- |
| Mental Health Disorder | Type 1 Diabetes (reference: no diabetes) | **1.14 (1.10, 1.17) ^a^** | **1.05 (1.04, 1.07) ^a^** | **1.02 (1.01, 1.03) ^a^** | **0.97 (0.96, 0.99) ^a^** |
|  | Type 2 Diabetes (reference: no diabetes) | **1.32 (1.28, 1.35) ^a^** | **1.24 (1.22, 1.26) ^a^** | **1.12 (1.11, 1.12) ^a^** | **1.03 (1.02, 1.04) ^a^** |
|  | Neuropathy (reference: none) | **1.51 (1.32, 1.73)** | **1.48 (1.42, 1.54)** | **1.55 (1.52, 1.57)** | **1.39 (1.37, 1.41)** |
|  | Retinopathy (reference: none) | **1.21 (1.08, 1.36)** | **1.11 (1.07, 1.15)** | **0.95 (0.93, 0.96)** | **0.90 (0.88, 0.92)** |
|  | Nephropathy (reference: none) | **1.23 (1.11, 1.36)** | **1.06 (1.02, 1.10)** | **1.14 (1.13, 1.16)** | **1.23 (1.22, 1.25)** |
|  | Myocardial Infarction (reference: none) | 1.45 (0.80, 2.61) | **1.38 (1.21, 1.56)** | **1.23 (1.20, 1.26)** | **1.23 (1.21, 1.25)** |
|  | Stroke (reference: none) | 1.17 (0.83, 1.64) | **1.58 (1.38, 1.81)** | **1.56 (1.52, 1.60)** | **1.46 (1.44, 1.48)** |
|  | Peripheral Vascular Disease (reference: none) | **1.39 (1.18, 1.65)** | **1.25 (1.18, 1.33)** | **1.22 (1.20, 1.23)** | **1.30 (1.29, 1.32)** |
|  | Amputation (reference: none) | **1.41 (1.12, 1.76)** | **1.10 (1.02, 1.19)** | **1.08 (1.05, 1.11)** | **1.17 (1.15, 1.19)** |
| Cognitive Disorder | Type 1 Diabetes (reference: no diabetes) | NA | NA | **0.93 (0.88, 0.98)** | **0.94 (0.92, 0.95)** |
|  | Type 2 Diabetes (reference: no diabetes) | NA | NA | **0.98 (0.94, 1.02)** | **0.93 (0.92, 0.94)** |
|  | Neuropathy (reference: none) | NA | NA | **1.76 (1.67, 1.84)** | **1.15 (1.13, 1.16)** |
|  | Retinopathy (reference: none) | NA | NA | 0.93 (0.86, 1.00) | **0.78 (0.76, 0.80)** |
|  | Nephropathy (reference: none) | NA | NA | **1.39 (1.32, 1.46)** | **1.39 (1.38, 1.41)** |
|  | Myocardial Infarction (reference: none) | NA | NA | **1.31 (1.20, 1.43)** | **1.29 (1.26, 1.31)** |
|  | Stroke (reference: none) | NA | NA | **3.08 (2.88, 3.28)** | **2.14 (2.10, 2.17)** |
|  | Peripheral Vascular Disease (reference: none) | NA | NA | **1.50 (1.43, 1.59)** | **1.57 (1.55, 1.59)** |
|  | Amputation (reference: none) | NA | NA | **1.12 (1.03, 1.22)** | **1.31 (1.28, 1.34)** |
| Chronic Pain | Type 1 Diabetes (reference: no diabetes) | **1.06 (1.03, 1.09) ^a^** | **1.03 (1.02, 1.05) ^a^** | **0.98 (0.97, 0.99) ^a^** | **0.95 (0.94, 0.96) ^a^** |
|  | Type 2 Diabetes (reference: no diabetes) | **1.45 (1.41, 1.49) ^a^** | **1.34 (1.33, 1.36) ^a^** | **1.13 (1.12, 1.13) ^a^** | **1.05 (1.05, 1.06) ^a^** |
|  | Neuropathy (reference: none) | **2.22 (1.91, 2.59)** | **1.71 (1.64, 1.79)** | **1.73 (1.71, 1.76)** | **1.62 (1.60, 1.64)** |
|  | Retinopathy (reference: none) | **1.27 (1.14, 1.42)** | **1.10 (1.06, 1.13)** | 0.98 (0.97, 1.00) | **0.97 (0.96, 0.99)** |
|  | Nephropathy (reference: none) | **1.12 (1.01, 1.24)** | **1.06 (1.02, 1.10)** | **1.14 (1.12, 1.15)** | **1.35 (1.34, 1.36)** |
|  | Myocardial Infarction (reference: none) | 1.78 (0.93, 3.42) | **1.29 (1.14, 1.46)** | **1.30 (1.26, 1.33)** | **1.39 (1.37, 1.42)** |
|  | Stroke (reference: none) | **1.96 (1.38, 2.80)** | **2.23 (1.91, 2.61)** | **1.71 (1.66, 1.76)** | **1.74 (1.71, 1.77)** |
|  | Peripheral Vascular Disease (reference: none) | **1.47 (1.24, 1.74)** | **1.45 (1.37, 1.53)** | **1.43 (1.41, 1.45)** | **1.44 (1.42, 1.45)** |
|  | Amputation (reference: none) | 1.23 (0.99, 1.53) | **1.10 (1.02, 1.19)** | **1.08 (1.05, 1.11)** | **1.23 (1.20, 1.25)** |
| Addiction | Type 1 Diabetes (reference: no diabetes) | 1.03 (0.97, 1.09) | **1.11 (1.08, 1.14) ^a^** | **0.91 (0.89, 0.92) ^a^** | **0.87 (0.85, 0.88) ^a^** |
|  | Type 2 Diabetes (reference: no diabetes) | **1.15 (1.09, 1.21)** | **1.25 (1.22, 1.28) ^a^** | **1.01 (1.00, 1.02) ^a^** | **0.96 (0.95, 0.97) ^a^** |
|  | Neuropathy (reference: none) | **1.54 (1.26, 1.88)** | **1.46 (1.38, 1.55)** | **1.44 (1.41, 1.46)** | **1.29 (1.26, 1.31)** |
|  | Retinopathy (reference: none) | **1.34 (1.10, 1.62)** | 0.98 (0.93, 1.03) | **0.80 (0.78, 0.82)** | **0.85 (0.83, 0.88)** |
|  | Nephropathy (reference: none) | **1.41 (1.19, 1.66)** | **1.11 (1.05, 1.17)** | **1.12 (1.10, 1.14)** | **1.14 (1.12, 1.15)** |
|  | Myocardial Infarction (reference: none) | 2.19 (0.71, 6.77) | **1.64 (1.41, 1.91)** | **1.66 (1.61, 1.71)** | **1.33 (1.30, 1.36)** |
|  | Stroke (reference: none) | 1.36 (0.78, 2.36) | **1.45 (1.23, 1.72)** | **1.58 (1.53, 1.62)** | **1.24 (1.22, 1.27)** |
|  | Peripheral Vascular Disease (reference: none) | 1.16 (0.87, 1.55) | **1.15 (1.06, 1.25)** | **1.45 (1.42, 1.47)** | **1.41 (1.39, 1.43)** |
|  | Amputation (reference: none) | **1.51 (1.10, 2.09)** | **1.30 (1.17, 1.44)** | **1.12 (1.08, 1.15)** | **1.03 (1.00, 1.06)** |
| Sleep Disorder | Type 1 Diabetes (reference: no diabetes) | 0.98 (0.92, 1.04) **^a^** | **0.94 (0.91, 0.96) ^a^** | **0.96 (0.95, 0.98) ^a^** | 0.99 (0.98, 1.01) **^a^** |
|  | Type 2 Diabetes (reference: no diabetes) | **1.55 (1.47, 1.63) ^a^** | **1.40 (1.37, 1.43) ^a^** | **1.26 (1.25, 1.27) ^a^** | **1.13 (1.12, 1.15) ^a^** |
|  | Neuropathy (reference: none) | **2.10 (1.69, 2.62)** | **1.48 (1.40, 1.56)** | **1.51 (1.49, 1.54)** | **1.44 (1.42, 1.46)** |
|  | Retinopathy (reference: none) | 1.05 (0.83, 1.34) | **1.09 (1.03, 1.14)** | **0.96 (0.94, 0.98)** | 0.99 (0.97, 1.01) |
|  | Nephropathy (reference: none) | 1.20 (0.99, 1.45) | **1.15 (1.09, 1.21)** | **1.23 (1.21, 1.25)** | **1.26 (1.24, 1.27)** |
|  | Myocardial Infarction (reference: none) | 2.02 (0.63, 6.46) | **1.51 (1.29, 1.76)** | **1.22 (1.19, 1.25)** | **1.15 (1.13, 1.18)** |
|  | Stroke (reference: none) | **3.89 (1.83, 8.25)** | **1.41 (1.20, 1.66)** | **1.36 (1.32, 1.40)** | **1.22 (1.20, 1.24)** |
|  | Peripheral Vascular Disease (reference: none) | **1.53 (1.16, 2.03)** | **1.38 (1.28, 1.49)** | **1.26 (1.24, 1.28)** | **1.20 (1.18, 1.21)** |
|  | Amputation (reference: none) | 1.43 (0.99, 2.06) | **1.14 (1.03, 1.27)** | **1.03 (1.00, 1.06)** | **1.03 (1.01, 1.06)** |

OR= Odds ratio, CI= confidence interval

**Bold Confidence intervals show a statistically significant different from their reference group**

^a^ represents a significant difference in odds between individuals with type 1 diabetes and individuals with type 2 diabetes
